# Supplementary material for: Mitochondria mediate septin cage assembly to promote autophagy of Shigella
Source: EMBO Rep. 2016 Jun 3;17(7):1029–43. doi: 10.15252/embr.201541832 (PMC4931556; doi:10.15252/embr.201541832)
Supplement: Supplementary file 1 — Expanded View Figures PDF [file EMBR-17-1029-s001.pdf]

Expanded View Figures

Figure EV1. SEPT7 is required for *Shigella*-septin cage formation.

A HeLa cells stably expressing SEPT6-GFP were infected with *Shigella flexneri* for 4 h 40 min, fixed for confocal microscopy and labelled with antibody for endogenous SEPT7. The scale bar represents 1 μm.

B HeLa cells were infected with *S. flexneri*-mCherry for 4 h 40 min, fixed for 3D-SIM and labelled with antibodies to endogenous SEPT7. Inset images show SEPT7 assembly into helix-like structure around *S. flexneri*. The scale bar represents 1 μm. See also Movie EV4.

C HeLa cells were treated with control (CTRL) or SEPT7 siRNA for 72 h. Whole-cell lysate of siRNA-treated cells were immunoblotted for GAPDH or SEPT7 to show the efficiency of SEPT7 depletion. GAPDH was used as a loading control. siRNA-treated cells were infected with *S. flexneri* for 4 h 40 min and then fixed and labelled with antibodies to SEPT2, SEPT7 or SEPT9 for quantitative microscopy. Graphs represent the mean % ± SEM of *Shigella* inside SEPT2, SEPT7 or SEPT9 cages from at least three independent experiments per treatment. Student's *t*-test, ***$P < 0.001$.

D HeLa cells were treated with control (CTRL) or two SEPT7 (−1 or −2) siRNA for 72 h, and whole-cell lysates were immunoblotted for SEPT2, SEPT6, SEPT7, SEPT9 or SEPT11. GAPDH was used as a loading control. Graph represents the mean % ± SEM of the relative amount of protein quantified by densitometry from at least three independent experiments per treatment. Student's *t*-test, ***$P < 0.001$.

E HeLa cells were treated with control (CTRL) or SEPT7 siRNA for 72 h, and the transcription level of SEPT2, SEPT6, SEPT7 or SEPT9 was quantified by qRT–PCR. GAPDH was used as control. Graph represents the mean ± SEM of the relative expression of GAPDH, SEPT2, SEPT6, SEPT7 or SEPT9 mRNA from two independent experiments per treatment. Student's *t*-test, ns = non-significant; **$P < 0.01$.

F HeLa cells were infected with x-light *Shigella* for 3 h 40 min or 4 h 40 min for quantitative confocal microscopy. IPTG was added 30 min prior to fixation, and then samples were labelled with antibody for SEPT7. Graph represents mean % ± SEM of *Shigella* responding to IPTG inside SEPT7 cages from at least three independent experiments per time point. Student's *t*-test, ns = non-significant.

 © 2016 The Authors

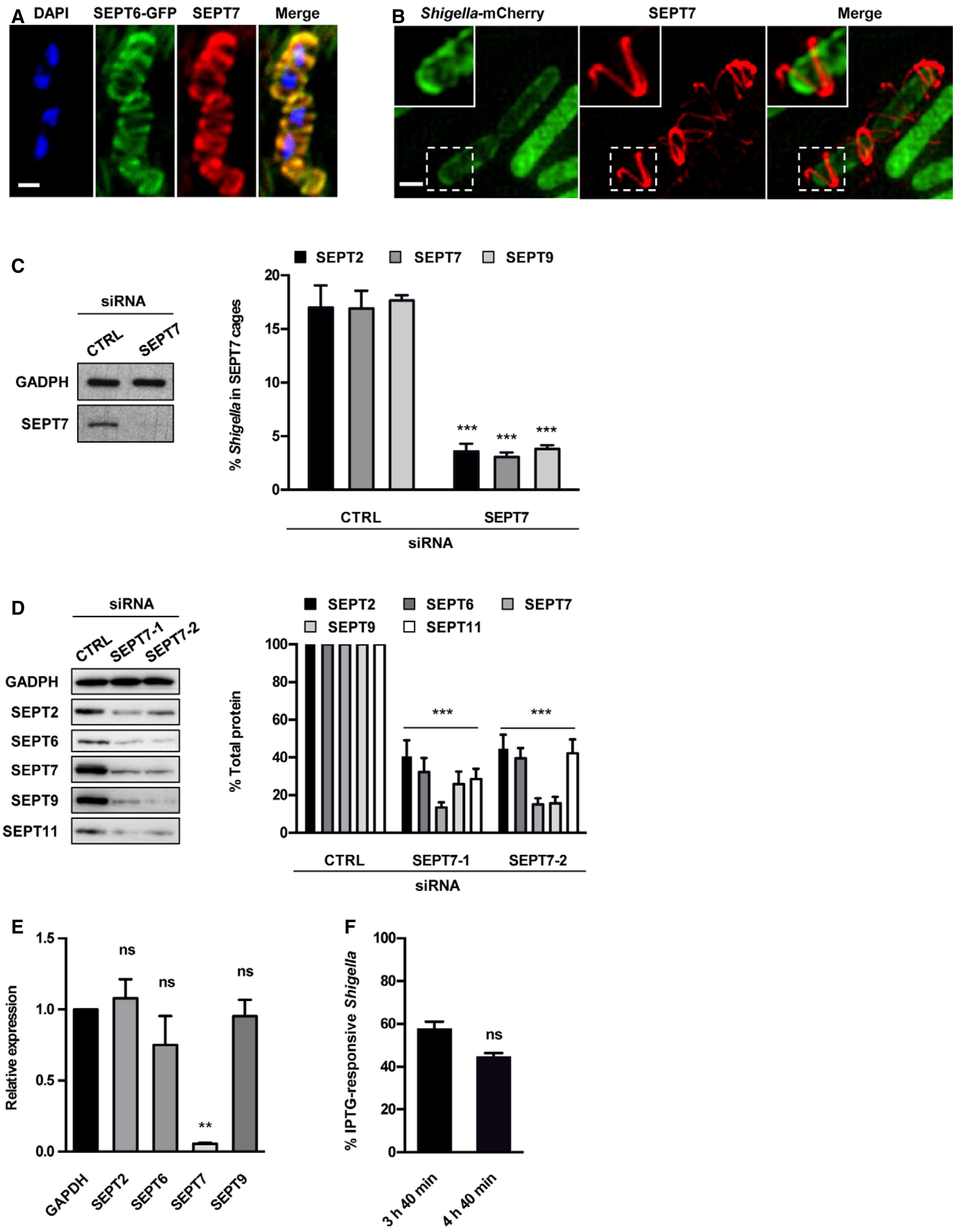
A
DAPI
SEPT6-GFP
SEPT7
Merge
B
Shigella-mCherry
SEPT7
Merge
C
siRNA
CTRL
SEPT7
GADPH
SEPT7
SEPT2
SEPT7
SEPT9
% Shigella in SEPT7 cages
20
15
10
5
0
***
***
***
CTRL
SEPT7
siRNA
D
siRNA
CTRL
SEPT7-1
SEPT7-2
GADPH
SEPT2
SEPT6
SEPT7
SEPT9
SEPT11
SEPT2
SEPT6
SEPT7
SEPT9
SEPT11
% Total protein
100
80
60
40
20
0
***
***
CTRL
SEPT7-1
SEPT7-2
siRNA
E
Relative expression
1.5
1.0
0.5
0.0
ns
ns
**
ns
GAPDH
SEPT2
SEPT6
SEPT7
SEPT9
F
% IPTG-responsive Shigella
100
80
60
40
20
0
ns
3 h 40 min
4 h 40 min

Figure EV1.

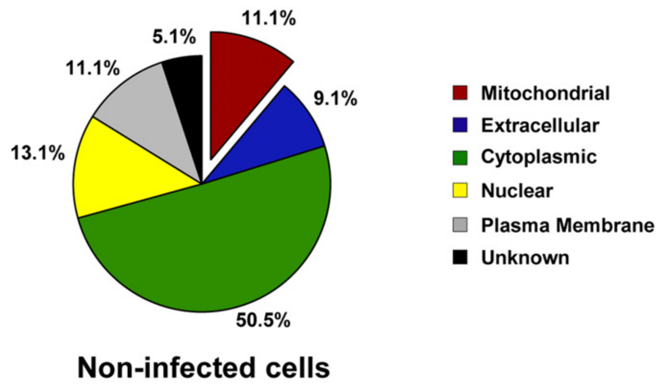

Figure EV2. Proteins associated with septins in uninfected cells.
The protein pulldown experiments identified 99 proteins putatively associated with the septin cytoskeleton in uninfected cells and then categorised into six groups based on the analysis from the Gene Ontology database. See also Dataset EV2.

Figure EV3. Mitochondria support septin assembly into rings and cages.

A HeLa cells were stained with MitoTracker Red CMXRos, treated with cytochalasin D for 30 min and then fixed and labelled for endogenous SEPT7 for confocal microscopy. Inset images highlight mitochondria closely associated with septin rings. The scale bar represents 5 μm.

B HeLa cells stably expressing SEPT6-GFP were transfected with mito-BFP for 24 h, infected with *Shigella*-mCherry for 4 h 40 min and processed for CLEM. Z-stack (Z1–Z4) series of septin cages were acquired by fluorescent light microscopy (FLM), and then samples were processed for TEM. SEPT6 is shown in green, mitochondria in red and *Shigella* in blue. The Z-stack (Z1–Z4) series clearly shows the septin-compartmentalised autophagosome and the mitochondrial membrane. The scale bar represents 5 μm.

C HeLa cells were treated with control (CTRL) or Mfn1 siRNA for 72 h, labelled with MitoTracker Red CMXRos, fixed for confocal microscopy and labelled with antibodies to Mfn1. The scale bar represents 5 μm. Inset images highlight Mfn1 associated with fused mitochondria fusion in CTRL cells, or absence of Mfn1 associated with fragmented mitochondria in Mfn1-depleted cells.

 © 2016 The Authors

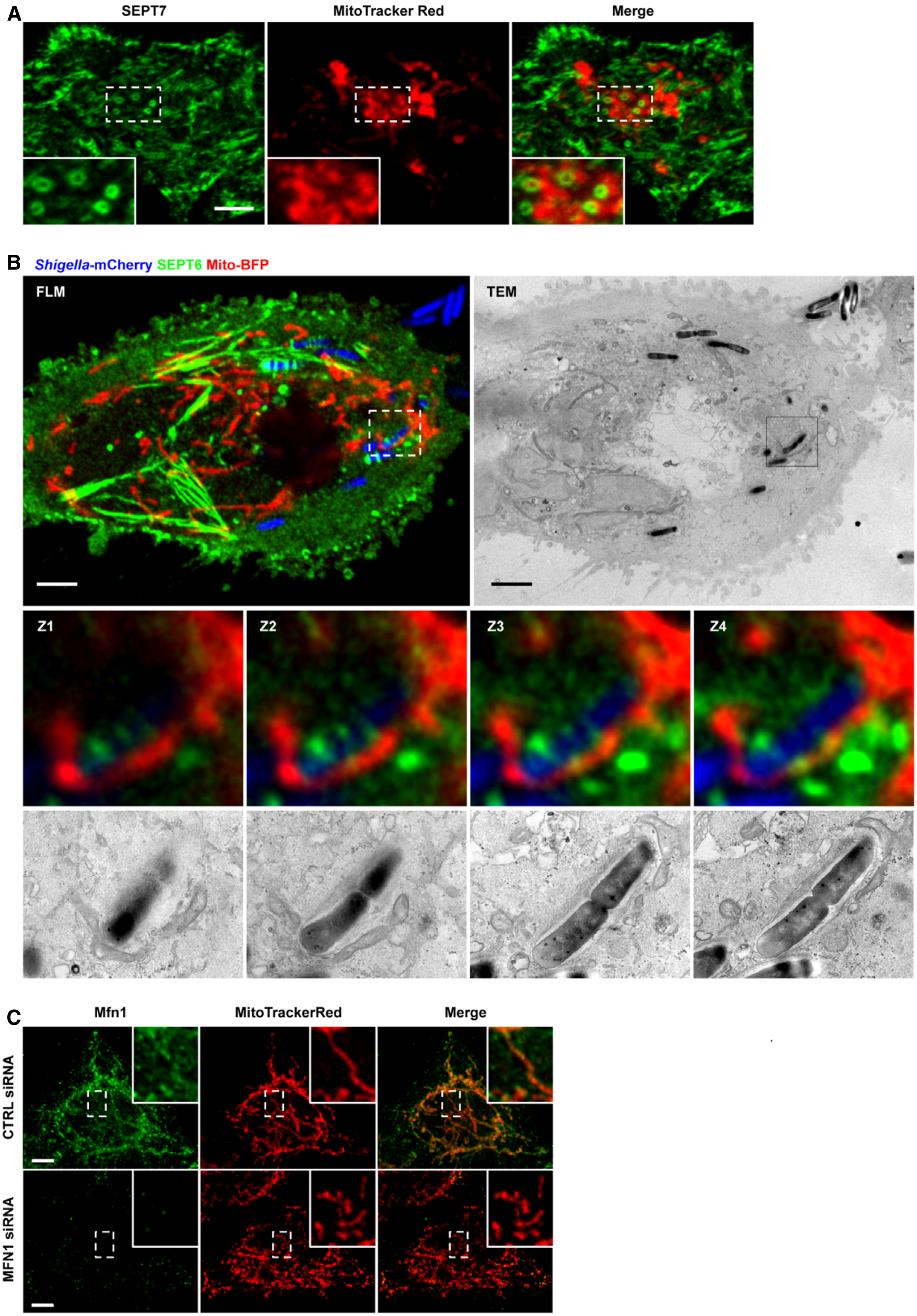
A
SEPT7
MitoTracker Red
Merge
B
Shigella-mCherry SEPT6 Mito-BFP
FLM
TEM
Z1
Z2
Z3
Z4
C
Mfn1
MitoTrackerRed
Merge
CTRL siRNA
MFN1 siRNA

Figure EV3.

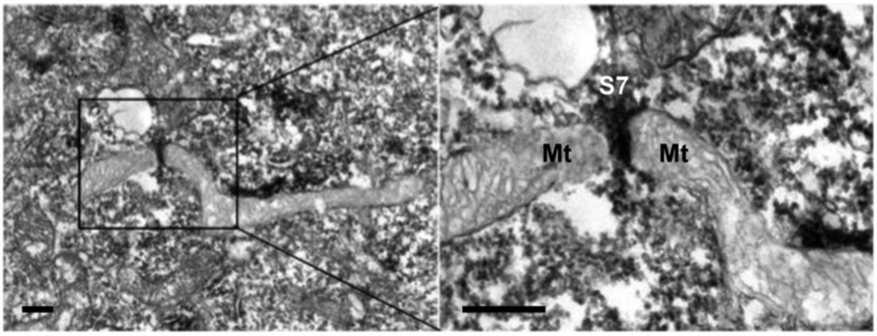

Figure EV4. Septin filaments intersect with sites of mitochondrial fission.
HeLa cells were fixed and labelled with antibody against SEPT7 and secondary antibody coupled to HRP and processed for TEM. The right image is enlarged from the boxed region in the TEM image and shows a SEPT7 (S7) filament (labelled in black) intersecting with the mitochondrial (Mt) fission site. The scale bar represents 100 nm.

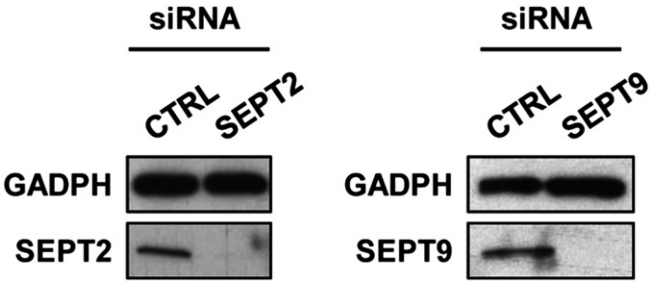

Figure EV5. Efficiency of SEPT2 or SEPT9 depletion by siRNA.
HeLa cells were treated with control (CTRL) or SEPT2 or SEPT9 siRNA for 72 h. Whole-cell lysate of siRNA-treated cells were immunoblotted for GAPDH, SEPT2 or SEPT9 to show efficiency of SEPT2 or SEPT9 depletion. GAPDH was used as a loading control.

 © 2016 The Authors
